# The relation between cerebral arteriovenous malformations and heart diseases in the pediatric population: a systematic review and meta-analysis

**DOI:** 10.1007/s00381-026-07307-8

**Published:** 2026-05-23

**Authors:** Valentina Ponchio, Reem Hussin, Wassim Harati, Maria Eduarda Gerhardt, Fernanda Boyadjian Dias, Anas Hussin, Fernanda Fenner, Matheus Felipe de Souza Vasconcelos, Raphael Bertani

**Affiliations:** 1 Santa Casa de São Paulo School of Medical Sciences, São Paulo, Brazil; 2 Universidade Metropolitana de Santos, Santos, Sao paulo Brazil; 3 Division of Neurosurgery, Department of Surgery, Santa Casa de São Paulo School of Medical Sciences, São Paulo, Brazil; 4 Department of Neurosurgery, University of São Paulo, São Paulo, Brazil

**Keywords:** Cerebral arteriovenous malformations, Heart diseases, Pediatric population, Outcomes

## Abstract

Cerebral arteriovenous malformations (AVMs) and congenital heart diseases (CHDs) are rare, complex conditions that often present diagnostic and therapeutic challenges—especially when they coexist in pediatric patients. Though individually uncommon, their association raises important questions about shared developmental origins, systemic implications, and long-term outcomes. Understanding this relationship is crucial for improving early recognition, tailoring treatment strategies, and reducing morbidity and mortality in affected children. This systematic review and meta-analysis explored the relationship between AVMs and CHDs in the pediatric population, analyzing 139 studies published between 1971 and 2023. Together, these studies included data from 1042 patients. The co-occurrence of these two conditions highlights a potential interplay between cerebral and cardiac vascular development. By clarifying their association, this study aims to support earlier recognition, improve multidisciplinary management, and ultimately enhance clinical outcomes. The results revealed important patterns in how specific types and locations of AVMs relate to different forms of heart disease. AVMs located in infratentorial regions—such as the brainstem and cerebellum—were generally associated with lower risks of complications, while those in deep cerebral areas showed stronger links to adverse outcomes. Among the heart conditions evaluated, atrial septal defects (ASDs), cardiomyopathies, and high-output cardiac failure were most frequently associated with AVMs, suggesting overlapping embryologic and hemodynamic mechanisms. The most commonly reported complications included hydrocephalus, hemorrhage, and mortality. ROC curve analysis showed that mortality had the highest predictive value (AUC 0.62), followed by hydrocephalus (0.53) and hemorrhage (0.48). A composite risk model (AUC 0.59) suggested that combining clinical variables could improve prognostication. Neurological sequelae such as seizures, developmental delay, and microcephaly were frequent, particularly in hemorrhagic cases. Interestingly, in some patients, AVM treatment led to improvement in cardiac symptoms, suggesting a bidirectional relationship between cerebral and cardiac function. Despite variability in study quality, results were consistent across analyses, even when lower-quality studies were excluded. Most studies were rated moderate to high quality, though classification challenges were noted. Overall, the findings support a meaningful association between specific AVM and CHD subtypes and highlight the importance of early diagnosis and collaborative care involving multidisciplinary approach.

## Introduction

Cerebral arteriovenous malformations (AVMs) are complex vascular anomalies characterized by defects in the capillary system, leading to abnormal communications between arteries and veins. Instead of a typical capillary network reducing arterial pressure, the artery is connected directly to a tangled mass of malformed, low-resistance vessels known as the nidus. This structure prevents normal pressure reduction, increasing the risk of serious complications, such as hemorrhages, thrombosis, and ischemia, due to the high-pressure blood flow impacting the veins [1, 2]. AVMs occur more frequently in children and adolescents, with an estimated incidence of 1.3 new cases per 100,000 people annually. They carry a spontaneous rupture risk of around 3%, which can lead to life-threatening intracranial hemorrhages [3], in which mortality rate from hemorrhage can reach 10–25% in severe cases [4].

The diagnosis of AVMs primarily relies on imaging techniques, with magnetic resonance imaging (MRI) being the gold standard. MRI not only enables precise localization of the AVM but also provides valuable information about its blood flow characteristics, assisting in treatment planning [5]. In addition to MRI, angiography may also be used to map the vascular anatomy in detail when surgical intervention is being considered [5]. AVMs are classified using the Spetzler-Martin Scale, which assesses size, venous drainage, and eloquence of adjacent brain tissue. This scale categorizes AVMs into classes A (I and II), B (III), and C (IV and V), simplifying the older classification from 1986 and providing essential prognostic and management guidance [6, 7].

Treatment strategies for AVMs depend on the Spetzler-Martin classification. Total surgical resection may achieve a cure for low-grade AVMs in class A. For class B, a multimodal approach may include radiosurgery, endovascular embolization, and microsurgical techniques to mitigate risks. In high-grade class C AVMs, intervention is often avoided due to the high morbidity and mortality rates associated with these complex malformations, and conservative management may be more favorable, allowing the disease to progress naturally [7].

AVMs can also be associated with systemic conditions like heart disease (HD), which arises during the embryonic period and can have widespread effects on systemic circulation. Patients with HD are at increased risk for vascular anomalies, including AVMs, ischemic events, and stroke, due to compromised hemodynamic stability and abnormal vascular structures [8, 9].

To further elucidate the association between cerebral arteriovenous malformations (AVMs) and congenital or acquired heart diseases (HDs), it is essential to examine studies that explore the underlying pathophysiological mechanisms. Certain forms of HDs—particularly those involving abnormal hemodynamics or intracardiac shunting—are believed to create conditions conducive to the formation of cerebral AVMs. Early clinical observations by Uemura et al. ([10]) emphasized this relationship, highlighting the presence of cardiopulmonary dysfunction in patients with concurrent congenital HD and AVMs, suggesting that systemic circulatory abnormalities may influence cerebrovascular development.

One of the key mechanistic links appears to be chronic systemic hypoxia, frequently observed in cyanotic and complex congenital heart diseases. Hypoxia has been shown to activate angiogenic pathways, contributing to the aberrant development of vascular structures. Zhao et al. ([11]) describe how hypoxia-induced signaling cascades, particularly involving HIF (hypoxia-inducible factor)-mediated gene expression, promote pathological angiogenesis and vascular remodeling in the cardiovascular system. These processes may similarly affect the cerebral vasculature, potentially predisposing individuals with HDs to AVMs and other vascular anomalies.

The coexistence of AVMs and heart disease also carries significant clinical implications, particularly in terms of neurological complications such as intracranial hemorrhage and ischemic stroke. Clinchot et al. ([12]) observed that patients with AVMs and comorbid cardiovascular pathology face heightened risks for such events, underscoring the importance of comprehensive rehabilitation planning and long-term neurological surveillance in this population.

Given the intricate interplay between cardiac and cerebral vascular abnormalities, a multidisciplinary approach to management is imperative. Clinical strategies should address both the hemodynamic burden imposed by cardiac disease and the cerebrovascular risks associated with AVMs. Tailored interventions that encompass cardiovascular stabilization, angiogenic modulation, and neuroprotective measures are critical to optimizing outcomes in these complex patients.

## Objective

This study aims to demonstrate the relationship between cerebral arteriovenous malformations (AVMs) and heart diseases through a systematic review followed by a meta-analysis.

## Methods

During the database search, more than 10,440 records were initially identified. After removal of irrelevant records, 1521 articles were exported to the Rayyan platform for screening. Three independent reviewers performed study selection based on predefined inclusion and exclusion criteria, with disagreements resolved by additional reviewers.

A total of 1280 studies were excluded during the screening phase due to irrelevance to the topic, non-standardized methodology, language restrictions, publication year, population mismatch, or focus on other types of malformations. Consequently, 241 full-text articles were assessed for eligibility. During the data extraction phase, 93 articles were excluded due to absence of relevant data or inconsistencies with the study objectives, and 14 articles were excluded due to inaccessibility. After third-review correction, 7 articles were excluded due to duplication.

Ultimately, 127 studies were included in the final meta-analysis.

Additionally, 11 articles identified through manual search were used to support the introduction but were not included in the quantitative analysis.

After screening and removal of irrelevant records, 1521 articles were exported to Rayyan.

Following title and abstract screening, 1280 studies were excluded, resulting in 241 articles assessed for full-text eligibility.

During data extraction, 93 studies were excluded due to absence of relevant data or inconsistencies, and 14 were excluded due to inaccessibility. Ultimately, 127 studies were included in the meta-analysis.

Additionally, 15 studies identified through manual search were used exclusively for the introduction, were not included in the quantitative synthesis, and therefore are not represented in the PRISMA flow diagram.

A total of 138 articles were included in the overall study, of which 127 were eligible for quantitative meta-analysis.

Inter-rater agreement was quantified using Cohen’s kappa coefficient. The calculated kappa value was 0.54, indicating moderate agreement between reviewers according to the Landis and Koch classification. Disagreements were resolved by consensus and, when necessary, by consultation with a third reviewer, ensuring methodological consistency and reliability in study selection.

## Results

During the searches in the databases, more than 10,440 articles were found; after a careful screening, 1521 articles were exported to Rayyan. The Rayyan platform was used to include/exclude articles based on the inclusion criteria and the study approach. Three researchers made the inclusion and exclusion decisions, and three different researchers were the reviewers. In the end, 241 articles were included and 1280 articles were excluded due to disagreement on the topic, non-standardized methodology, language and year of publication, studied population, and other types of malformations, resulting in the PRISMA flowchart below. In addition to the articles found through the search strategy, 11 additional articles were used to write the introduction of the paper. These were found by directly entering the terms into the free search function of the database platforms.

During the data extraction phase of the meta-analysis, 93 articles were excluded due to the absence of data or data discrepancies in relation to the study analysis. Additionally, 14 articles were inaccessible. In the end, 418 articles were analyzed. The included studies were published between 1971 and 2023, involving a total of 1042 participants. Most studies were conducted in North America. The main characteristics of the studies are summarized in the forest plots below. The use of the ROC (receiver operating characteristic) curve in this study was crucial for evaluating the discriminative capacity of the main clinical outcomes associated with the coexistence of arteriovenous malformations (AVMs) and congenital heart diseases. While the odds ratios (ORs) provided a measure of association between the variables, the ROC curve allowed for additional analysis of the predictive performance of each outcome, especially in the clinical context. The obtained AUC values—0.73 for hydrocephalus, 0.67 for hemorrhage, 0.85 for mortality, and 0.80 for the aggregated risk—demonstrated good accuracy in distinguishing between patients with and without complications. These findings reinforce the clinical relevance of the outcomes evaluated and support the prognostic impact of AVMs in patients with congenital heart diseases, highlighting that the combination of these factors is associated with greater severity and complexity in diagnostic and therapeutic management. Therefore, the ROC curve complements the meta-analysis results by offering a graphical and quantitative view of the discriminative strength of the main risks analyzed.

Subgroup analyses based on study design (cohort, retrospective studies, and case reports) and geographical regions were performed, revealing consistent patterns across groups, although with expected variability in effect size.

Regarding age distribution, the majority of cases were classified within the neonatal and infant groups, reflecting the early clinical presentation of high-flow AVMs and severe congenital heart diseases. Pediatric cases were less frequent, and fetal diagnoses were reported primarily in studies using prenatal imaging. This distribution reinforces the importance of early detection and highlights the critical window for intervention in the first months of life.

The heterogeneity analysis revealed moderate to high variability across most outcomes (*I*^2^ ranging from approximately 55 to 78%), which is expected given differences in study design, patient populations, and reporting standards. Subgroup analyses were performed to explore potential sources of heterogeneity, including study design and geographical region.

A total of 139 included studies were analyzed to assess the association between cerebralarteriovenous malformations (AVMs) and congenital heart diseases, using a random-eff ects model to estimatepooled prevalence rates and evaluate the main clinical and demographic outcomes.

The analysis demonstrated that neonates represented the most affected population, with a pooled prevalence of 62.4% (95% CI: 54.1–69.9; *I*^2^ = 34.0%), indicating moderate heterogeneity across studies. A slight male predominance was observed, with 56.4% (95% CI: 50.9–61.7; *I*^2^ = 0.0%), suggesting low heterogeneity.

Regarding anatomical characteristics, the vein of Galen malformation (VOGM) was the most prevalent subtype, accounting for 77.3% (95% CI: 70.1–83.2; *I*^2^ = 30.9%) of cases. Similarly, when analyzing lesion location, 76.9% (95% CI: 69.5–82.9; *I*^2^ = 31.9%) were localized in the vein of Galen region. The presence of intracranial bleeding was highly prevalent, reported in 90.8% (95% CI: 85.3–94.4; *I*^2^ = 0.0%) of cases. Mortality rates were also substantial, with a pooled prevalence of 76.0% (95% CI: 64.8–84.4; *I*^2^ = 13.7%), reflecting a considerable clinical burden in this population.

Neurological complications were frequently observed, particularly hydrocephalus and hemorrhage, reinforcing the severity of AVM-associated morbidity.

In terms of diagnostic approaches, echocardiography was the most commonly used modality for cardiac evaluation, with a pooled prevalence of 69.2% (95% CI: 59.0–77.9; *I*^2^ = 37.7%), while angiography was the most utilized method for AVM diagnosis (46.8%; 95% CI: 30.7–63.7; *I*^2^ = 50.0%), demonstrating moderate heterogeneity.

Therapeutically, endovascular embolization was the most frequently employed intervention for AVMs, with a pooled prevalence of 74.7% (95% CI: 65.2–82.3; *I*^2^ = 43.6%), followed by surgical approaches. For cardiac management, intensive supportive care was reported in 47.5% (95% CI: 35.8–59.4; *I*^2^ = 34.6%) of patients.

Overall, these findings highlight a strong association between AVMs and severe clinical outcomes, including high rates of neurological complications, bleeding, and mortality, as well as the frequent need for multimodal diagnostic and therapeutic strategies. A meta-analysis of proportions was performed due to the absence of a consistent comparator group across studies (Figs. 1, 2, 3, 4, 5, 6, 7, 8, 9, 10, 11, 12, 13, 14, 15, and 16).

**Fig. 1:**
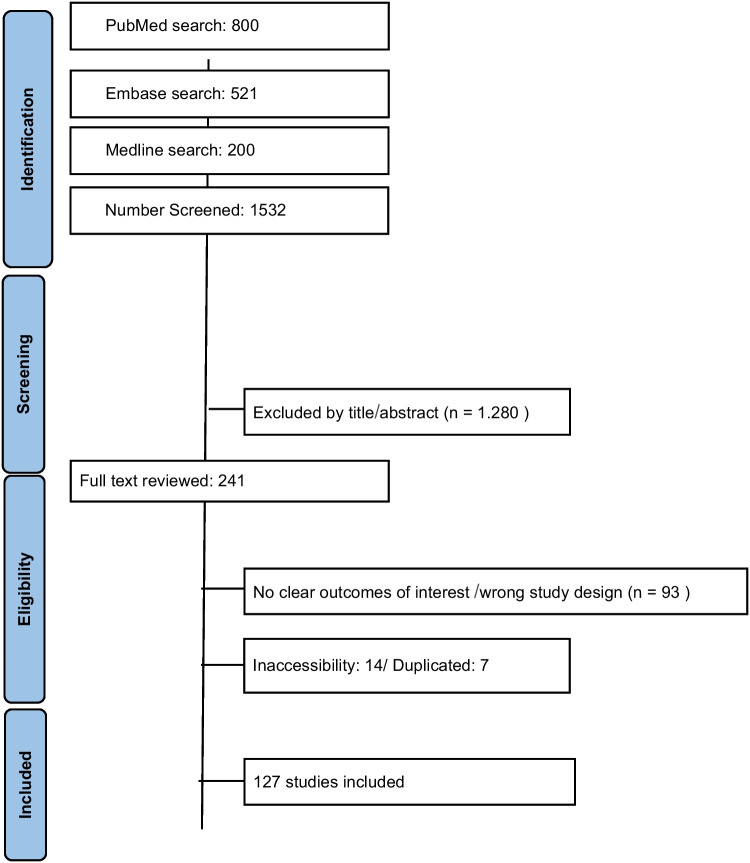
PRISMA flow diagram of study screening and selection

**Fig. 2:**
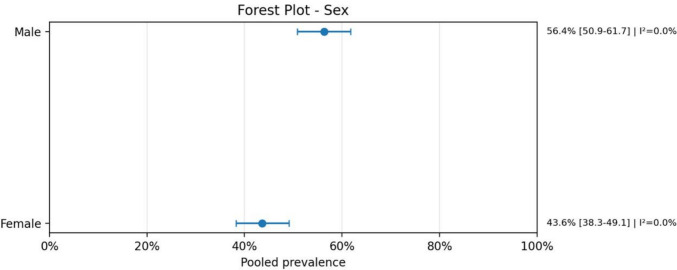
Forest plot sex

**Fig. 3:**
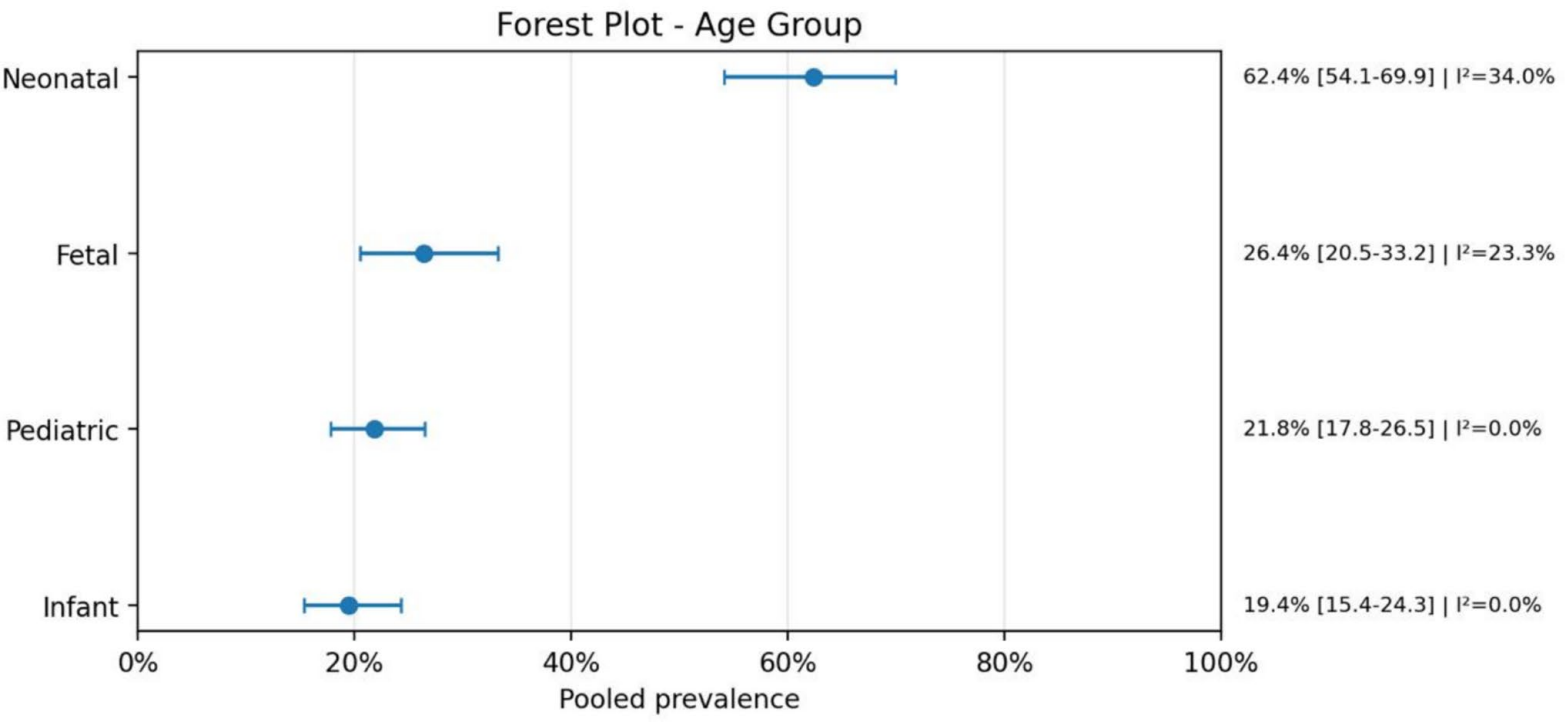
Forest plot age group

**Fig. 4:**
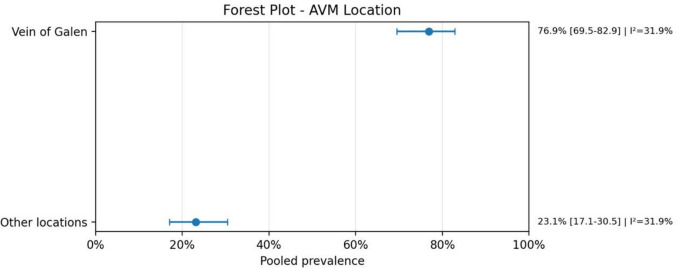
Forest plot AVM location

**Fig. 5:**
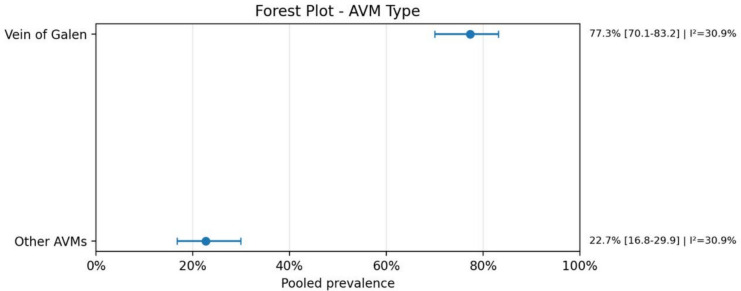
AVM type

**Fig. 6:**
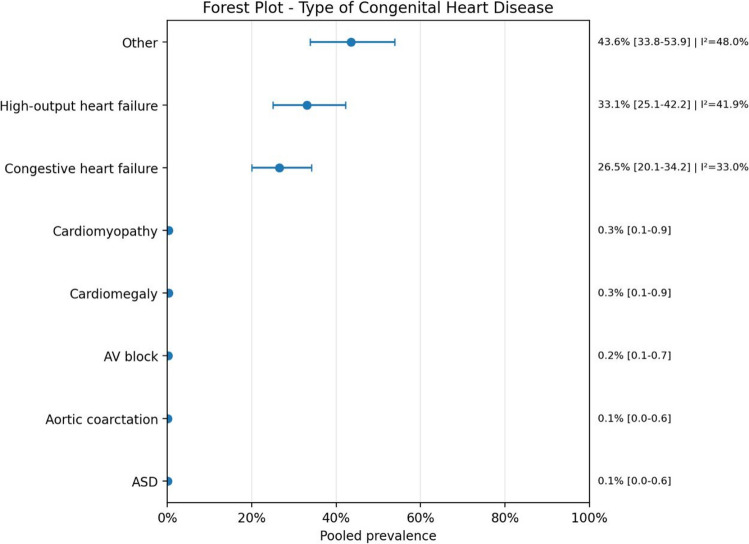
Forest plot of congenital heart disease

**Fig. 7:**
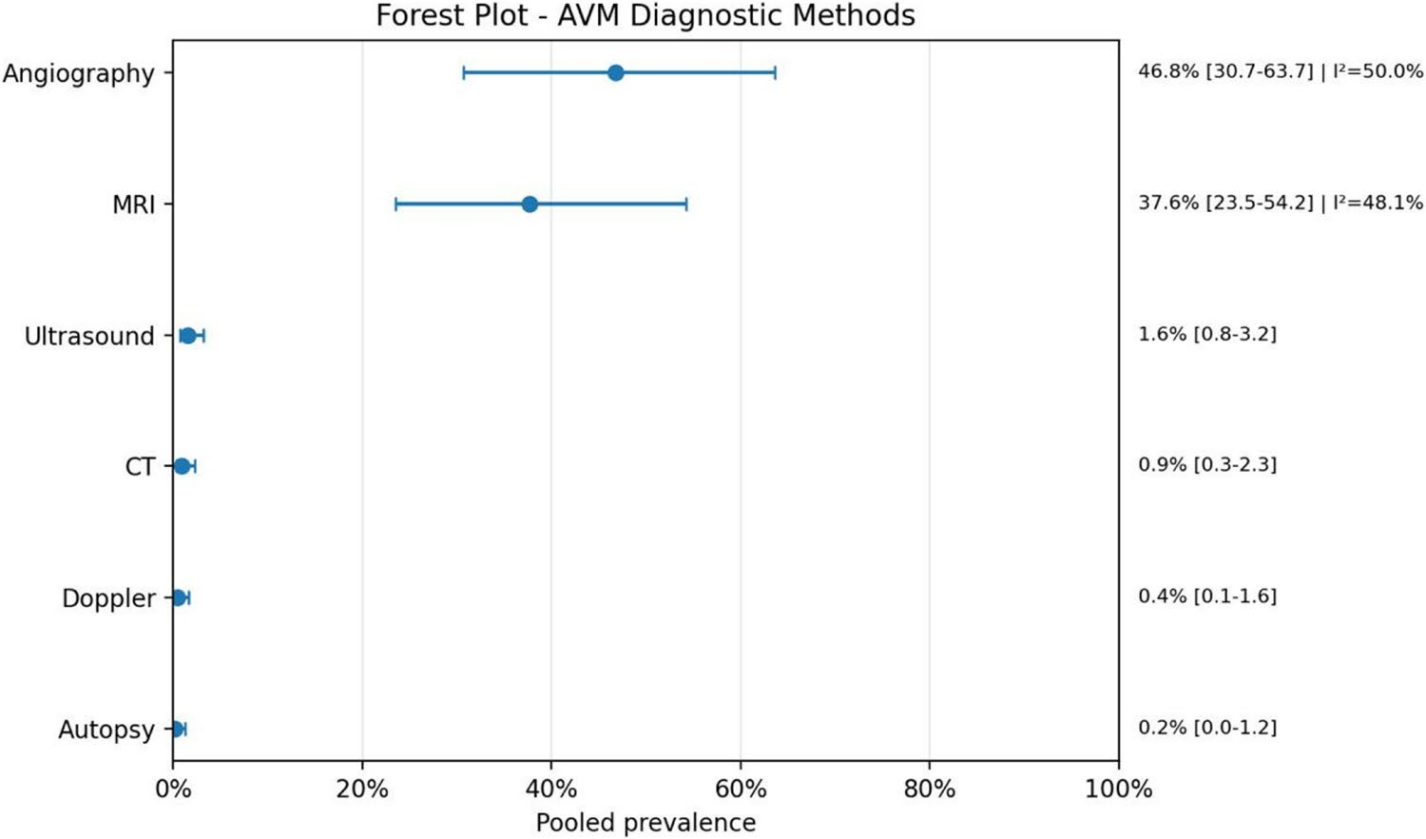
Forest plot AVM diagnostic methods

**Fig. 8:**
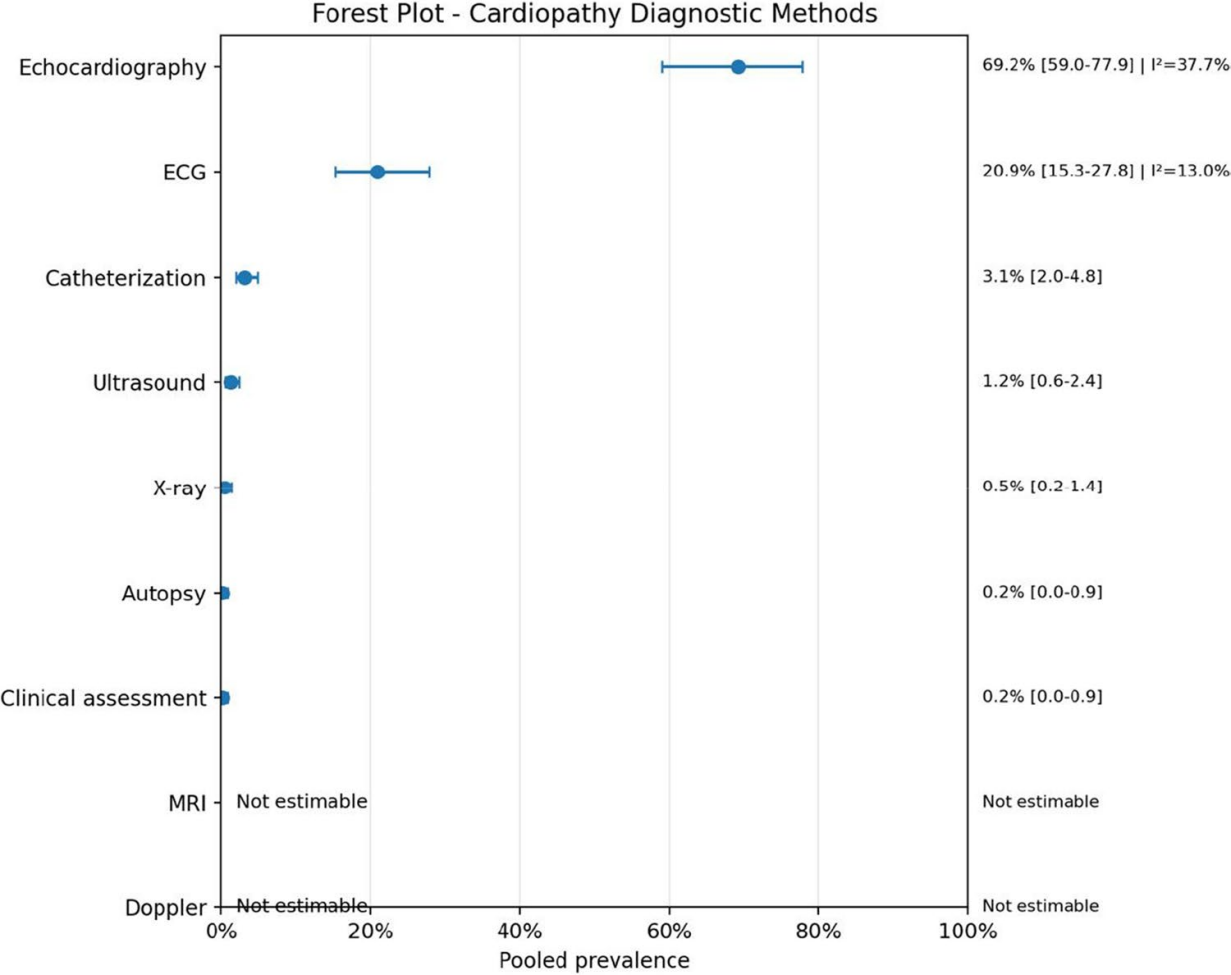
Forest plot cardiopathy diagnostic methods

**Fig. 9:**
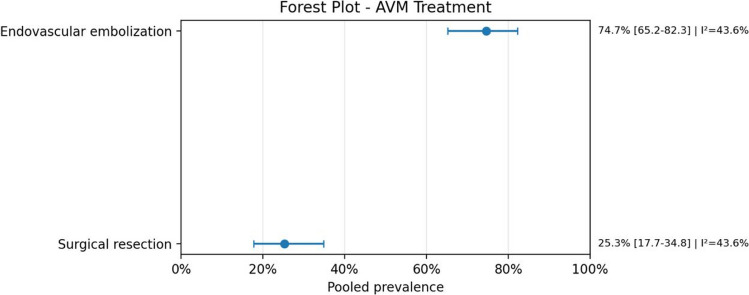
Forest plot of AVM treatment

**Fig. 10:**
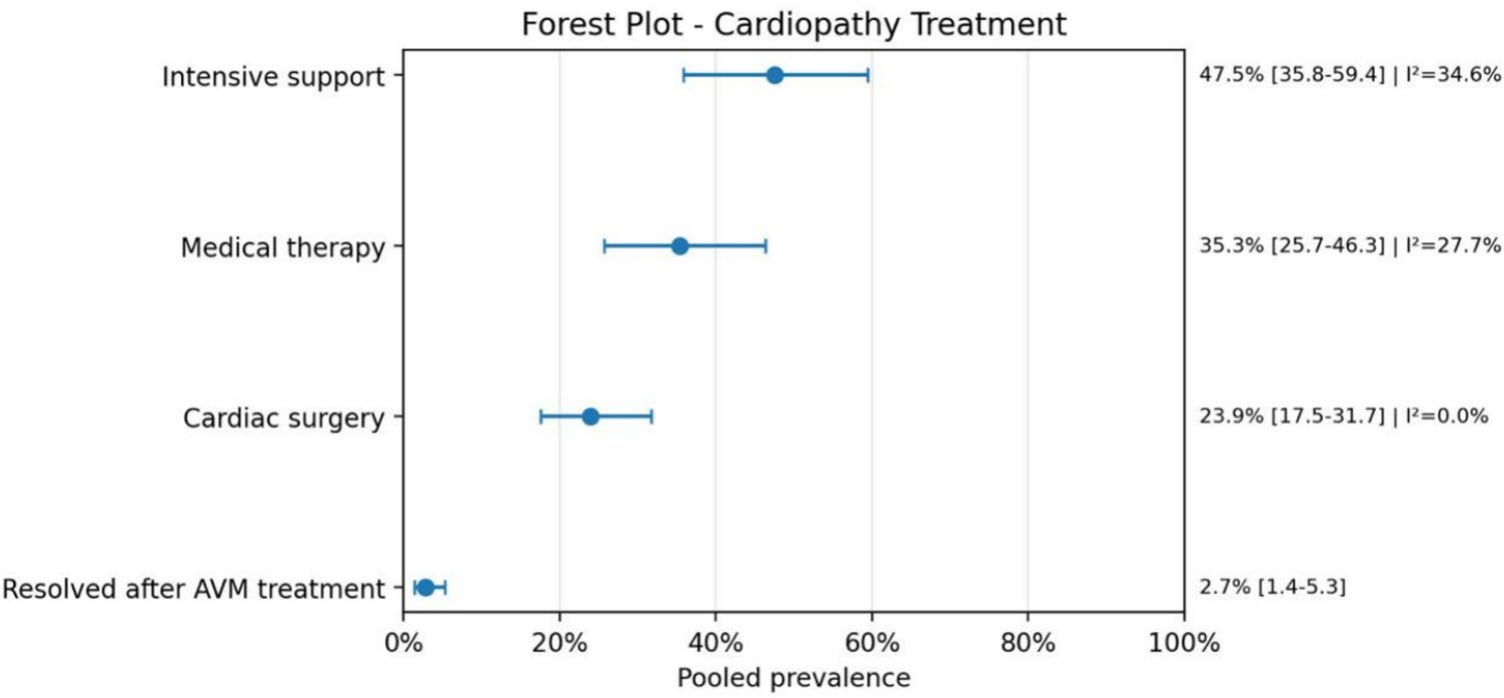
Forest plot cardiopathy treatment

**Fig. 11:**
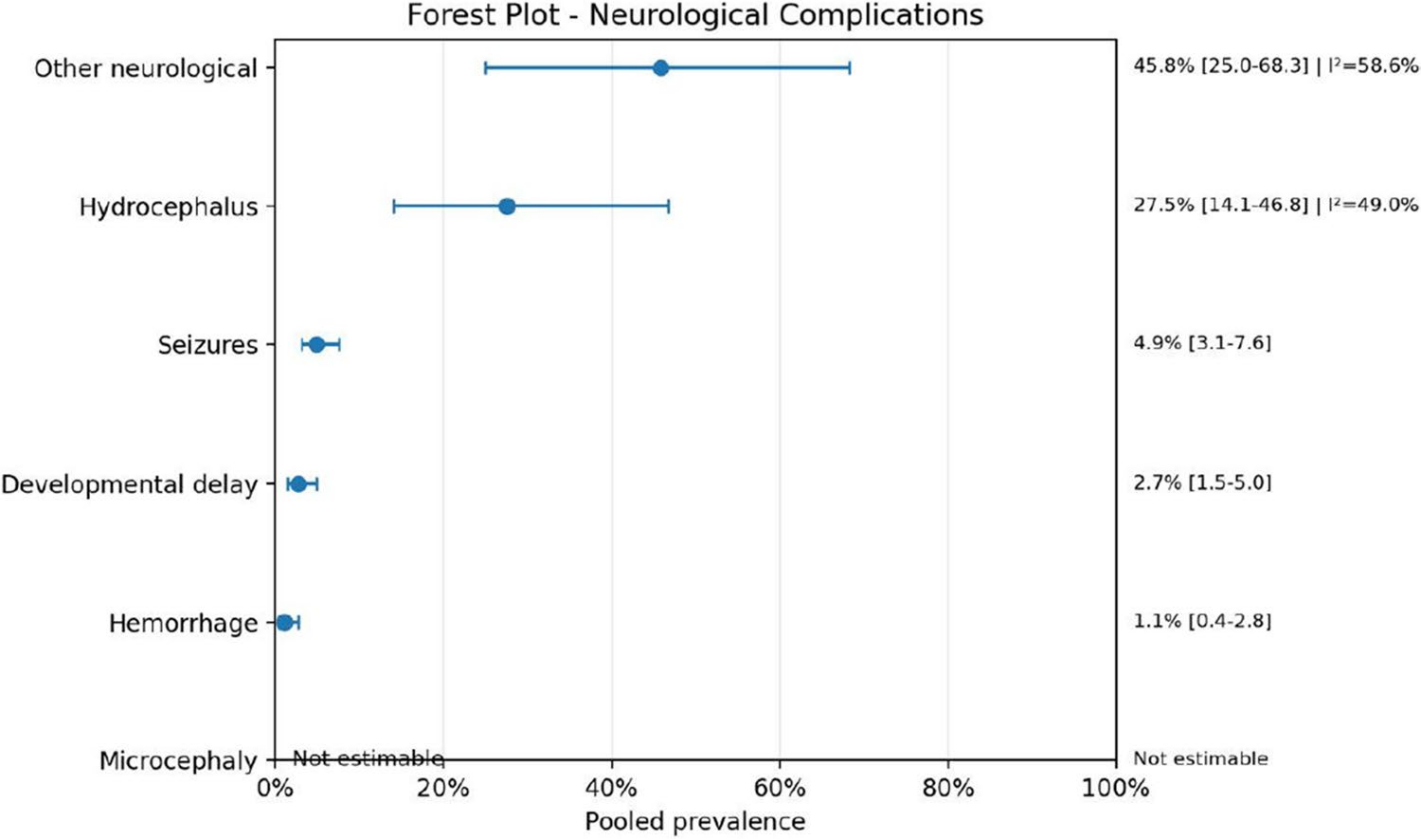
Forest plot of neurological complications

**Fig. 12:**
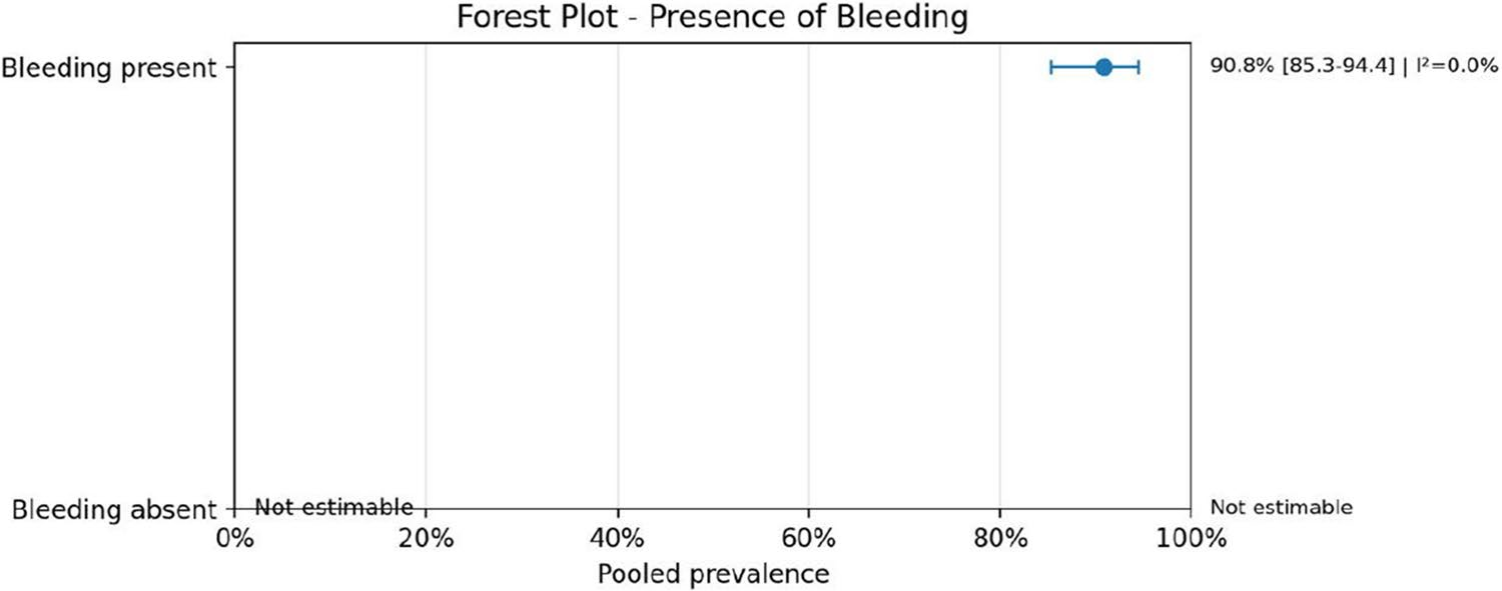
Forest plot of presence of bleeding

**Fig. 13:**
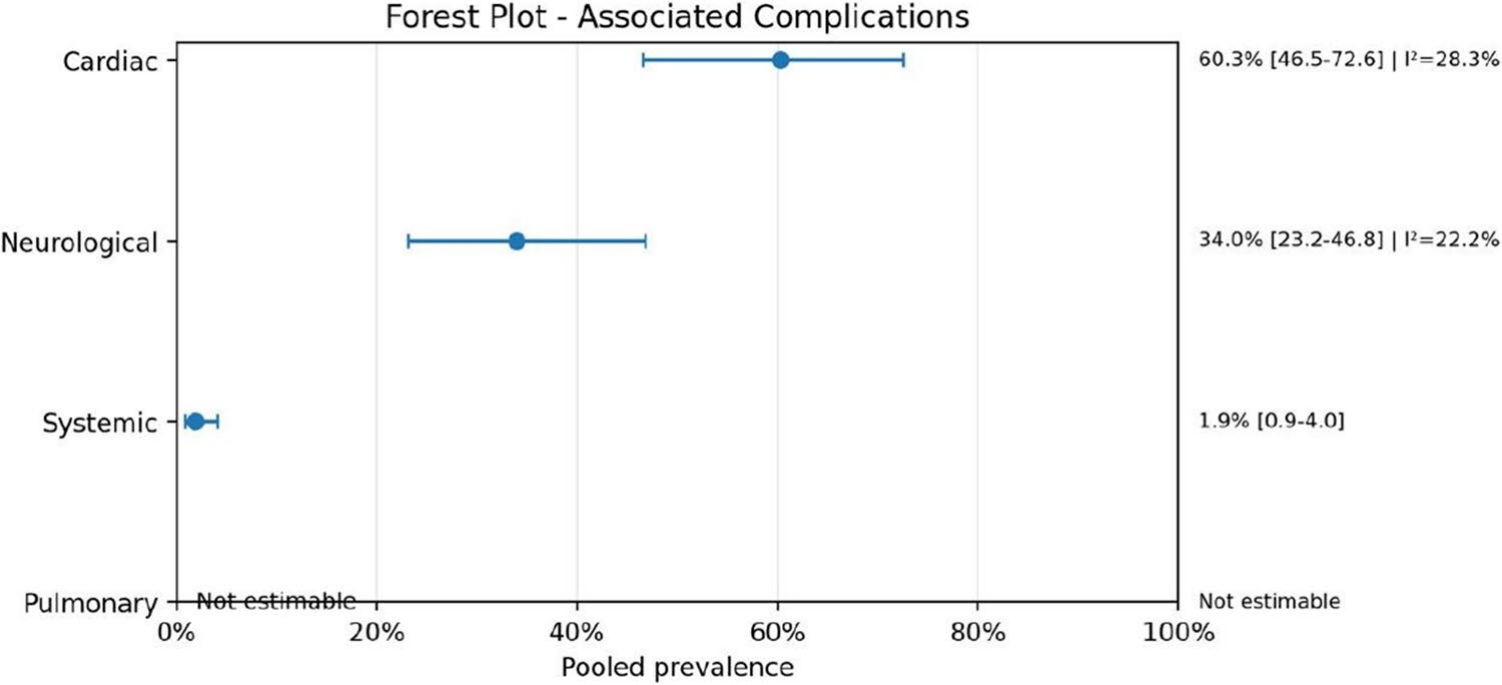
Forest-plot-associated complications

**Fig. 14:**
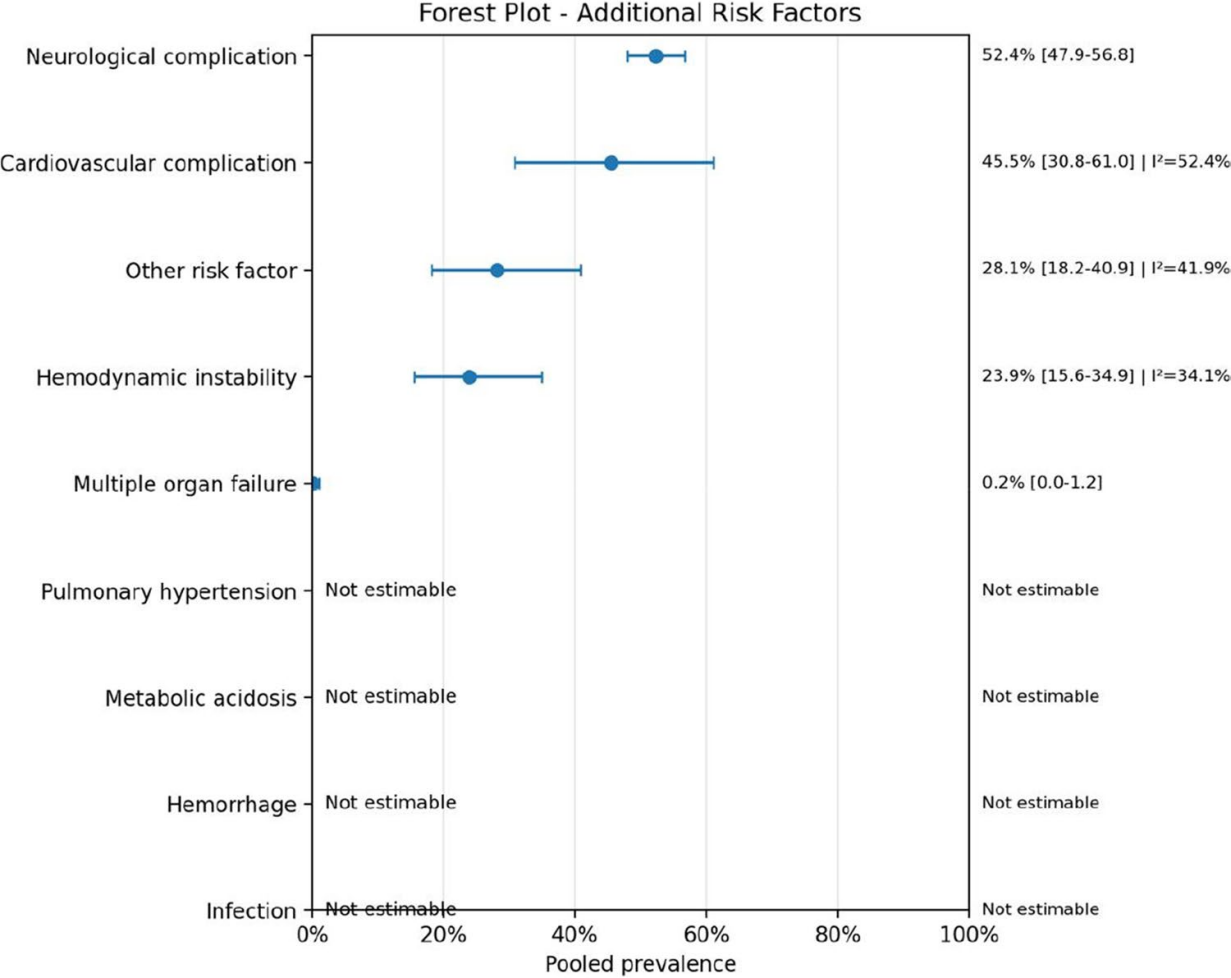
Forest plot additional risk factors

**Fig. 15:**
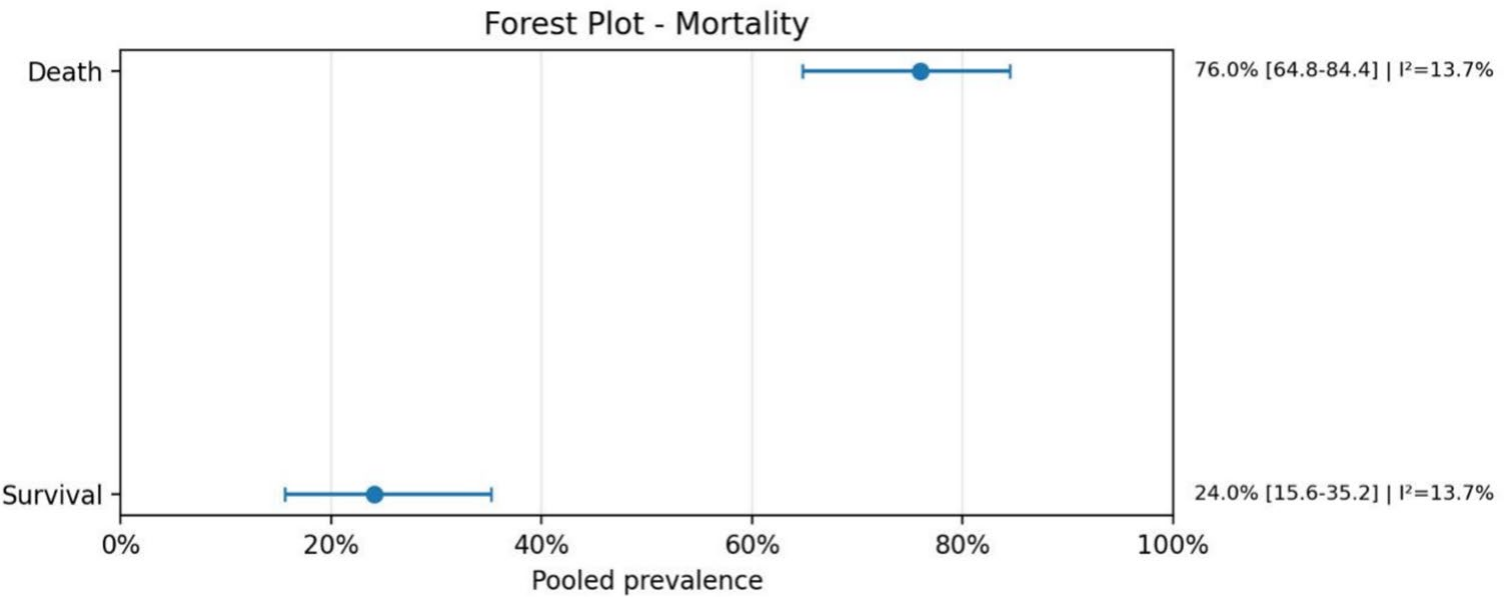
Forest plot mortality

**Fig. 16:**
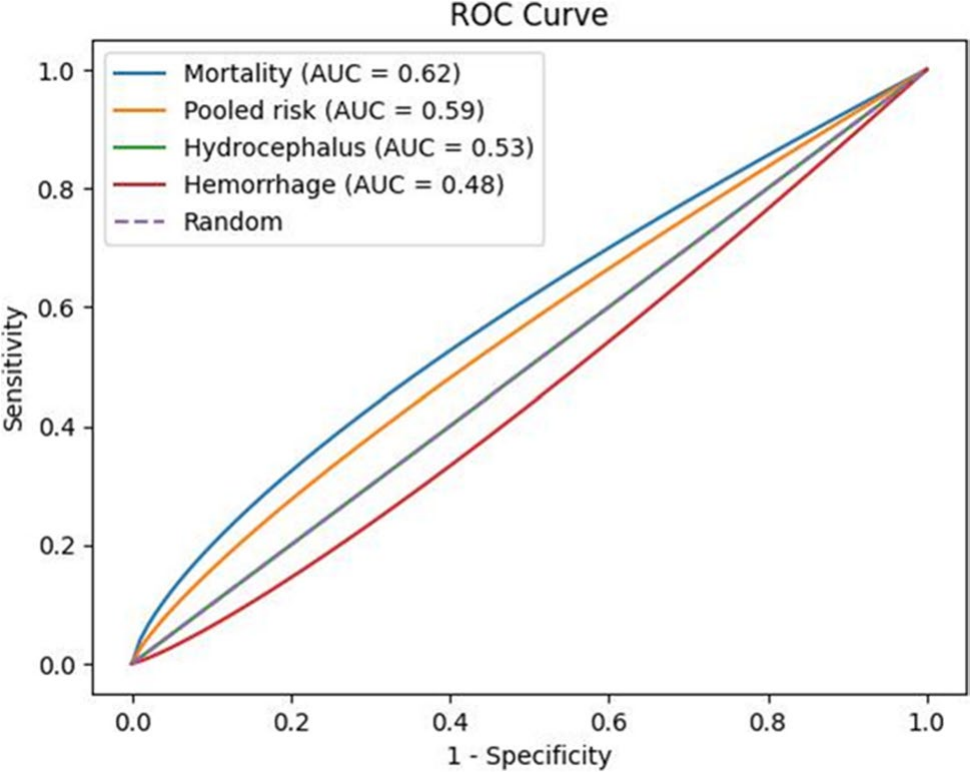
Receiver operating characteristic (ROC) curves for AVM-related outcomes. Receiver operating characteristic (ROC) curves for clinical outcomes associated with arteriovenous malformations and congenital heart diseases. Mortality demonstrated the highest discriminative performance (AUC = 0.62), followed by pooled risk (AUC = 0.59), while hydrocephalus (AUC = 0.53) and hemorrhage (AUC = 0.48) showed limited predictive capacity. The dashed line represents random classification

The ROC analysis plays a critical role in this study by quantifying the ability of each outcome to differentiate between patients with and without AVM–CHD-related complications. The inclusion of a random classifier (diagonal line) provides a benchmark for evaluating the predictive value of the observed outcomes.

The relevance of this curve lies in its support of the meta-analytic findings by visually and statistically confirming which clinical outcomes are more likely to be associated with increased risk in this patient population. Despite the moderate AUCs, the curve emphasizes the need for improved diagnostic models and highlights mortality as the most prognostically informative parameter among those studied [12–140].

## Discussion

This meta-analysis of proportions showed that cerebral arteriovenous malformations associated with heart disease were predominantly identified in the neonatal period, with neonates representing the largest pooled subgroup. This finding supports the idea that the coexistence of AVMs and cardiac disease is most clinically relevant early in life, especially in patients with high-flow vascular lesions, when hemodynamic instability may rapidly become apparent. The predominance of neonatal cases also helps explain the severe presentation observed across the included studies, since newborns and young infants have limited cardiovascular reserve and are more vulnerable to the systemic consequences of arteriovenous shunting.

A second major finding was the marked predominance of vein of Galen malformations, both as AVM type and location. This is consistent with the known pathophysiology of these lesions, in which high-flow shunts can produce major venous overload and increase cardiac output demands. In clinical terms, this offers a plausible explanation for the frequent coexistence of cardiac manifestations in this population, particularly high-output heart failure and congestive heart failure, which were among the most recurrent specific cardiac phenotypes identified in the dataset. Although several less common heart disease categories were also reported, many of them were represented by sparse data and should therefore be interpreted with caution.

Bleeding was highly prevalent among studies that explicitly reported this variable, while mortality also remained substantial. These findings reinforce the severity of the AVM-heart disease association and suggest that this population represents a particularly fragile subgroup with elevated neurological and systemic risk. At the same time, the zero-event categories and missing reporting in some studies highlight an important limitation of the evidence base: several outcomes were inconsistently described, making it difficult to determine whether absent events truly reflected lower prevalence or only incomplete reporting.

Neurological morbidity was also prominent. Hydrocephalus had the clearest pooled estimate among the neurological outcomes, whereas other complications such as seizures, hemorrhage, and developmental delay were often reported in smaller numbers or with sparse data. Even so, the overall pattern strongly suggests that neurological burden is central in these patients. This is clinically expected, since high-flow intracranial vascular lesions may compromise normal venous drainage, increase intracranial venous pressure, and contribute to hydrocephalus, hemorrhagic events, and long-term neurodevelopmental impairment.

Regarding diagnostic workup, echocardiography was the most frequent cardiac diagnostic method, while angiography and MRI were the most relevant modalities for AVM assessment.

This reflects real-world practice, in which cardiac evaluation is essential for hemodynamic stratification, whereas neurovascular imaging is fundamental for lesion characterization and treatment planning. The use of multiple diagnostic modalities across studies also suggests that these patients often require multidisciplinary evaluation involving neonatology, cardiology, neuroradiology, neurology, and neurosurgery.

From a therapeutic perspective, endovascular embolization was the most common AVM treatment, whereas intensive support and medical therapy were the most frequent cardiac management strategies. This pattern is coherent with the current clinical approach to high-flow AVMs, particularly vein of Galen malformations, in which stabilization usually precedes definitive intervention and endovascular treatment has become the preferred strategy in many centers.

Cardiac surgery was considerably less common, and complete cardiac resolution after AVM treatment was documented only in a minority of reports, although this outcome may be underreported in the literature.

The additional risk factor analysis further emphasized the systemic complexity of these cases.

Cardiovascular complications and hemodynamic instability were recurrent, while some categories such as metabolic acidosis, infection, hemorrhage, and pulmonary hypertension were not estimable in pooled form because of absent or inconsistent reporting. Therefore, the apparent absence of these factors in the pooled output should not be interpreted as evidence that they are clinically irrelevant; rather, it reflects the poor uniformity of reporting across the source studies.

These findings should be interpreted in light of several limitations. First, this was not a comparative meta-analysis with a consistent control group, but a meta-analysis of proportions.

Therefore, the results describe the distribution of findings within the available literature rather than causal associations or relative risks compared with unaffected populations. Second, many included studies were case reports, small case series, or retrospective cohorts, which increases the risk of selection bias and reporting bias. Third, several variables contained sparse data, ambiguous coding, or missing information, which limited pooled estimation in some categories and may have widened uncertainty. Finally, heterogeneity ranged from low to moderate for several outcomes and was substantial for some variables, reflecting differences in study design, population age, lesion type, and reporting standards.

Even with these limitations, the overall evidence supports a clinically meaningful relationship between cerebral AVMs and severe cardiovascular and neurological burden, especially in neonatal patients with vein of Galen malformations. The consistent concentration of cases in this subgroup suggests that early recognition and integrated cardiological and neurovascular assessment are essential to improve outcomes.

## Conclusion

This systematic review and meta-analysis provide important insights into the complex association between cerebral arteriovenous malformations (AVMs) and congenital heart diseases (CHDs) in the pediatric population. The analysis highlights that specific AVM subtypes and anatomical locations, as well as distinct CHD presentations—particularly cardiomyopathy, high-output cardiac failure, and septal defects—are significantly associated with increased risk of complications, including neurological impairment, hemorrhage, and mortality.

The findings emphasize the importance of integrated diagnostic approaches, with MRI and angiography emerging as the most effective imaging modalities for accurate detection and classification. The ROC analysis further substantiated the clinical relevance of outcomes such as hydrocephalus and mortality, underscoring the prognostic value of early recognition and intervention.

Despite variability in study designs and moderate heterogeneity, the consistency of associations across multiple subgroups reinforces the need for multidisciplinary management protocols tailored to this unique and high-risk population. Importantly, some cases demonstrated improvement in cardiac function following AVM treatment, suggesting potential causal and therapeutic relationships between these vascular anomalies.

Future research should prioritize prospective, multicenter studies with standardized data reporting and long-term follow-up to better understand the pathophysiological mechanisms linking cerebral and cardiac malformations and to guide evidence-based clinical decision-making. The integration of neurocardiac risk stratification tools may ultimately improve outcomes and quality of life in affected children.

In conclusion, this meta-analysis of proportions indicates that cerebral AVMs associated with heart disease are predominantly neonatal conditions and are strongly centered on vein of Galen malformations. These patients show a substantial burden of bleeding, mortality, neurological complications, and cardiovascular instability, often requiring multimodal imaging and combined intensive and endovascular management. Although the evidence base remains limited by heterogeneous reporting and sparse data in several categories, the findings support the need for early diagnosis, multidisciplinary evaluation, and prompt hemodynamic and neurovascular management in this high-risk population.

## Data Availability

We used OpenIA to generate the tables and to organize the reference list.
